# ^18^F-FDG-PET Imaging for Post-COVID-19 Brain and Skeletal Muscle Alterations

**DOI:** 10.3390/v13112283

**Published:** 2021-11-15

**Authors:** Thorsten Rudroff, Craig D. Workman, Laura L. Boles Ponto

**Affiliations:** 1 Department of Health and Human Physiology, University of Iowa, E432 Field House, Iowa City, IA 52242, USA; craig-workman@uiowa.edu; 2 Department of Neurology, University of Iowa Hospitals and Clinics, Iowa City, IA 52242, USA; 3 Department of Radiology, University of Iowa Hospitals and Clinics, Iowa City, IA 52242, USA; laura-ponto@uiowa.edu

**Keywords:** post-COVID, PET, glucose uptake, brain, muscle, hypometabolism

## Abstract

Scientific evidence concerning the subacute and long-term effects of coronavirus disease 2019 (COVID-19) is on the rise. It has been established that infection by serious acute respiratory syndrome coronavirus 2 (SARS-CoV-2) is a systemic process that involves multiple organs. The complications and long-term consequences of COVID-19 are diverse and patients need a multidisciplinary treatment approach in the acute and post-acute stages of the disease. A significant proportion of COVID-19 patients experience neurological manifestations, some enduring for several months post-recovery. However, brain and skeletal muscle changes resultant from SARS CoV-2 infection remain largely unknown. Here, we provide a brief overview of the current knowledge, and usefulness, of [^18^F]fluorodeoxyglucose positron emission tomography/computed tomography (^18^F-FDG-PET/CT) to investigate brain and skeletal muscles changes in Post-COVID-19 patients with persistent symptoms. Furthermore, a brief discussion of future ^18^F-FDG-PET/CT applications that might advance the current knowledge of the pathogenesis of post-COVID-19 is also provided.

## 1. Introduction

As of 1 November 2021, there have been 246,594,191 confirmed cases of coronavirus disease 2019 (COVID-19) subsequent to severe acute respiratory syndrome coronavirus 2 (SARS-CoV-2) infection, resulting in 4,998,784, deaths globally (https://covid19.who.int/, accessed on 3 November 2021). Most knowledge on COVID-19 focuses almost entirely on the acute illness and symptoms, such as cough, fever, myalgia, ageusia and anosmia [1,2,3]. However, the reality of the long-term consequences of COVID-19 is becoming increasingly more obvious [4,5]. Indeed, many survivors of COVID-19 have chronic post-viral complications similar to the previous severe acute respiratory syndrome (SARS) and Middle East respiratory syndrome pandemics [6]. A generally accepted standardized clinical case definition of this post-viral state is lacking [7]. Various terminologies and definitions, including long COVID, COVID long-haulers, post-acute COVID-19, late sequelae of COVID-19, or post-COVID-19 have been proposed and controversies still exist about the correct naming. In this narrative mini-review, we will use the terminology “post-COVID-19”, as recommended by the World Health Organization (WHO). The WHO defines post-COVID-19 as a condition that occurs in individuals with a history of probable or confirmed SARS-CoV-2 infection, usually 3 months from the onset of COVID-19, with symptoms that last for at least 2 months and cannot be explained by an alternative diagnosis [8]. Furthermore, WHO lists fatigue, shortness of breath, and cognitive dysfunction [8] among common symptoms which generally have an impact on activities of daily living.

Fatigue is the most common post-COVID-19 symptom, with a prevalence ranging from 17.5% to 72% among hospitalized patients and can endure for more than seven months after the onset of illness in many cases [5,9,10,11,12,13,14,15]. Other common post-COVID-19 symptoms, which might also last for several months and disrupt work activities and quality of life, include olfactory and gustatory dysfunction, dyspnea, myalgia, chest pain, and mental health and sleep disorders [5,16,17,18,19].

Scientific evidence of the persistence of neurological symptoms following acute COVID-19 is increasing. It is a process recently termed Neuro-PASC (neurological manifestations of post-acute sequelae of SARS-CoV-2 infection) [20]. Numerous COVID-19 patients suffer from PASC, with the number of cases severely increasing as more people are infected [21]. However, it is still unclear how SARS-CoV-2 results in pathological changes in the CNS [22]. Two main hypotheses for the causes of Neuro-PASC are a) indirect effects via peripheral inflammation or b) direct effects via SARS-CoV-2 CNS invasion. Regarding the former, Mehta et al. [23] postulated that a cytokine storm (i.e., an inflated immune response instigated by SARS-CoV-2 infection) might play an indirect role in these neurological manifestations of PASC [23]. On the other hand, some reports also suggest that SARS-CoV-2 may directly invade the CNS, possibly infecting brain cells via the functional receptor human angiotensin-converting enzyme 2 (hACE2). However, hACE2 is minimally present in the brain and evidence of the SARS-CoV-2 infection has been infrequently reported in cerebral spinal fluid analyses [1,2]. The occurrence of this is greater in patients needing hospitalization, especially those in the Intensive Care Unit [23,24,25]. However, despite large variability in persistent symptomatology, the most commonly reported neurologic manifestations are fatigue, “brain fog”, headache, numbness/tingling, dysgeusia, anosmia, and myalgia [26,27]. Studies have also shown that PASC can impact children, young adults, and those who only had mild COVID-19 symptoms and did not require respiratory support or hospitalization [15,28].

Questions regarding the mechanisms underlying the pathophysiology of PASC symptoms in post-COVID-19 patients are still unanswered. Fontana et al. [29] proposed that positron emission tomography (PET) imaging might significantly contribute to our understanding of the pathophysiological brain changes in post-COVID-19 patients by identifying the affected brain regions and the involved cell types. Furthermore, the underlying mechanisms can be explored by investigating changes in neurotransmission or metabolic parameters. ^18^F-FDG-PET/CT can be a useful tool to detect or rule out severe concomitant processes. Alterations of brain metabolism shown with this technique may be a marker of the systemic process [7]. However, a review of literature focused on amalgamating the results of PET scans in post-COVID-19 patients has not been undertaken. Therefore, the purpose of this mini-narrative review was to integrate the results of PET studies in post-COVID-19 patients. The focus was on investigations that employed the [^18^F]fluorodeoxyglucose (^18^F-FDG) radiotracer, a fluorine-18 radiolabeled glucose analog, which is the most common PET radiotracer worldwide. A record search of the PubMed and Scopus databases was performed to identify publications between 1 January 2020 and 31 October 2021, that applied positron emission tomography to people with post-COVID symptoms. The search terms included “PET” or “positron emission tomography” AND “Long-COVID” or “Long-COVID 19” or “Post-COVID” or “Post-COVID-19” and were the same for both databases. The search results were screened and selected by two assessors. Relevant studies not detected by literature search were added manually. This narrative mini-review followed the WHO’s definition of Post-COVID-19 but included studies with COVID-19 survivors with persistent symptoms after at least three weeks since infection.

## 2. ^18^F-FDG-PET/CT as a Tool for Assessing Post-COVID-19 Brain Changes

Advantages of PET/CT include the ability to perform a whole-body assessment, high patient compliance, and a favorable safety profile. For many years, the cerebral ^18^F-FDG-PET signal was biologically interpreted as a direct index of neuronal activity [30]. Specifically, regional glucose consumption and, by extension, the ^18^F-FDG cerebral uptake, correlated with local neuronal and synaptic activity as neurotransmission and signal transduction has a high energetic requirement [31]. Connections between neurons are processed mainly by excitatory glutamatergic synapses which constitute the majority of all cortical synapses, yielding an energetic consumption of ~80% of total cortical glucose usage.

However, a more consolidative view in which astrocytes, an abundant glial cell, are also significant contributors to the ^18^F-FDG-PET signal was hypothesized [32]. Indeed, more recent evidence that astrocytes significantly contribute to the ^18^F-FDG-PET signal has emerged [33,34]. Moreover, it is well recognized that astrocytes play significant roles in the brain’s defense against peripheral inflammatory changes [35]. Specifically, the cells involved in the inflammatory response demonstrate greater glycolytic activity and the affinity of glucose transporters for deoxyglucose during inflammation appears to be increased by cytokines and growth factors [36,37]. Furthermore, increased glucose consumption/^18^F-FDG uptake might result from cellular stress induced by cell injury (i.e., metabolic flare) [36]. Hence, ^18^F-FDG-PET might be very useful to identify these areas of increased inflammation and infection in the investigation of post-COVID-19 symptoms.

## 3. ^18^F-FDG-PET/CT and Brain Metabolism Changes in Post-COVID-19 Patients

Some studies (summarized in Table 1) have shown that Post-COVID-19 patients with persistent functional symptoms and complaints demonstrate continuous ^18^F-FDG-PET hypometabolism in various brain regions [37,38,39,40,41,42,43]. Reduced metabolic activity in the orbitofrontal cortex in COVID-19 anosmia was found by Karimi-Galougahi et al. [37], which might suggest that impaired neural function of this region might be a causative mechanism for anosmia, likely due to direct neurotropism of SARS-CoV-2 [39]. Guedj et al. [38] analyzed ^18^F-FDG brain PET of post-COVID-19 patients with a biologically confirmed diagnosis of SARS-CoV-2 infection and persistent functional complaints at least 3 weeks after the initial infection. They found bilateral hypometabolism in the bilateral rectal/orbital gyrus, including the olfactory gyrus; the right temporal lobe, including the amygdala, hippocampus, and right thalamus; the bilateral pons/medulla brainstem; and the bilateral cerebellum. Importantly, this hypometabolism was associated with the patients’ symptoms (e.g., hyposmia/anosmia, memory/cognitive impairment, pain and insomnia). Sollini et al. [39] also demonstrated brain hypometabolism thalamus as well as the right parahippocampal gyrus in 13 post-COVID-19 patients that were associated with persistent symptoms (e.g., anosmia/ageusia and fatigue). Similarly, Donegani et al. [40] demonstrated relative hypometabolism in bilateral parahippocampal and fusiform gyri and in the left insula in post-COVID-19 patients with hyposmia compared to controls. This finding largely confirms the topography of brain hypometabolism in patients with post-COVID-19 with persistent hyposmia or with other functional complaints [41]. Lastly, Dressing et al. [43] assessed cognitive profiles and regional cerebral glucose metabolism as a biomarker of neuronal function in outpatients suffering from long-term neurocognitive symptoms after COVID-19. Patients with long-term symptoms (202 ± 58 days after positive PCR) were assessed with a neuropsychological test battery and cerebral ^18^F-FDG PET imaging was performed in a subset of the patients. Only mild impairments on neuropsychological testing and no significant findings on ^18^F-FDG PET were found.

Although less studied than the adult COVID-19 symptomology, one study has used ^18^F-FDG-PET to assess brain metabolism in pediatric patients. Morand et al. [42] investigated seven children at least 4 weeks after initial COVID-19 symptoms (e.g., fatigue, fever, chills) and found that, despite lower initial severity at the acute stage of the infection, children showed similar brain hypometabolism as adult post-COVID-19 patients, involving bilateral medial temporal lobes, brainstem and cerebellum. This study provides arguments in favor of possible post-COVID-19 in children detected by ^18^F-FDG-PET.

In summary, the ^18^F-FDG-PET evidence presented above implies a sensitivity of the frontal lobes, or at least of the frontal hubs of cortical-subcortical networks, to SARS-CoV-2 infection. Thus, an inflammatory parainfectious route preferentially targeting the frontal lobes (and/or frontal networks) might be underpinning these shared neurophysiological, clinical, and imaging findings in COVID-19 patients [44]. Furthermore, the current cerebral ^18^F-FDG-PET evidence seems to support the indirect, peripheral inflammation hypothesis of Neuro-PASC over direct CNS invasion. This might have been expected given that hACE2 expression is negligible in the brain and SARS-CoV-2 would have little intracellular ingress, as discussed above.

**Table 1:** Summary of the characteristics and findings of the ^18^F-FDG-PET studies included in the review.

Study	Design	N	Age (Years)	Sex (F/M)	Time Since COVID-19	Summary of Findings
Karimi-Galougahi et al. [37]	Case Study	1	27	1/0	6 weeks	Reduced metabolic activity in the orbitofrontal cortex.
Sollini et al. [39]	Case Control	13	54 (46–80)	5/8	132 ± 31 days	^18^F-FDG uptake in several “target” and “non-target” tissues, with a typical pattern of brain hypometabolism.
Guedj et al. [38]	Case Control	35	55 ± 11	20/15	>3 weeks	Hypometabolism involving the olfactory gyrus and connected limbic/paralimbic regions, extended to the left insula in patients with respect to controls.
Donegani et al. [40]	Cohort Study	22	64 ± 10.5	10/12	>1 month	Relative hypometabolism was demonstrated in bilateral parahippocampal and fusiform gyri and in left insula in patients with respect to controls.
Morand et al. [42]	Cohort Study	7	12 ± 2	6/1	1 month	Brain hypometabolic pattern in children, involving bilateral medial temporal lobes, brainstem, and cerebellum.
Dressing et al. [43]	Cohort Study	14	54 ± 2	N/A	>3 months	Cerebral ^18^F-FDG PET failed to reveal a distinct pathological signature in the subgroup of patients undergoing ^18^F-FDG PET.
Topuz et al. [45]	Cohort Study	68	56 ± 15	32/36	1 month	Increased SUVmax values obtained from the psoas muscle.

## 4. Long-Term Consequences of Brain Hypometabolism for Post-COVID-19 Patients

Interestingly, ^18^F-FDG brain hypometabolism in the pre-frontal cortex has also been associated with multiple neurodegenerative disorders [30] and neuropsychiatric conditions [46], sometimes even prior to the first clinical symptoms. For example, ^18^F-FDG-PET imaging has uncovered the characteristic glucose metabolic reductions in the frontal, parieto-temporal, and posterior cingulate (PCC) cortices in Alzheimer’s disease (AD). Generally, this pattern of cortical metabolic alterations has been beneficial for predicting a future AD diagnosis and in differentiating AD from other neurodegenerative diseases. ^18^F-FDG-PET measures of these cortical regions have previously provided a good partition of AD from neurologically healthy controls [47,48]. Specifically, Herholz et al. [49] evaluated 395 AD patients and 110 normal controls subjects and found that a decline in ^18^F-FDG uptake in the parieto-temporal, PCC, and prefrontal association cortices identified mild to moderate AD with 93% sensitivity and specificity, and very mild AD (i.e., mini-mental status examination (MMSE) ≥ 4) with 84% sensitivity and 93% specificity. A recent meta-analysis [50] also reported that ^18^F-FDG-PET sensitivity for AD vs. neurologically healthy controls ranged from 61% to 100%, and specificity from 54% to 100%. Additionally, a review by Mosconi [30] indicated that distinctive patterns of brain metabolism were associated with specific forms of dementia. Similarly, Lehrer et al. [46] reported relative metabolic rate reduction in the thalamus and lateral prefrontal cortex in patients with schizophrenia; importantly, these findings were from a patient group that had never taken medication, indicating that these reductions could not be due to medication artifacts. Taken together, these studies support PET as a powerful tool for discriminating diseased/disordered brains from neurologically healthy brains. Importantly, a very recent study by Hosp et al. [51] examined the neurological long-term effects of COVID-19 using ^18^F-FDG-PET. The scans indicated pathological results in 10/15 patients, predominantly presenting as frontoparietal hypometabolism. Furthermore, they verified these findings via comparison with a control group using voxel-wise principal components analysis and found a high correlation (r^2^ = 0.62) with the Montreal Cognitive Assessment. Furthermore, neocortical dysfunction with cognitive decline was revealed in a significant proportion of patients that required inpatient treatment at COVID-19 onset [51].

## 5. ^18^F-FDG-PET/CT and Skeletal Muscle Changes in Post-COVID-19 Patients

Neuromuscular manifestations, such as ophthalmoparesis, hyposmia/ageusia, facial paresis, neuropathy, Guillain-Barré syndrome, Myasthenia Gravis, myopathy, myalgia, myositis, and rhabdomyolysis can be investigated with whole-body ^18^F-FDG-PET/CT. Myalgia, for example, is described among the common symptoms of post-COVID-19 [52]; however, the underlying mechanisms are unclear. Skeletal muscles and cells within the muscles (e.g., leukocytes, satellite cells, endothelial cells, and fibroblasts) express angiotensin-converting enzyme 2 (ACE2), which interacts with SARS-CoV-2 in its spike domain and might make skeletal muscles vulnerable to direct virus invasion [53]. Other potential mechanisms are the release of myotoxic cytokines, immune complex deposition in muscles, damage from homology between viral antigens and human muscle cells, and absorption of viral protein on muscle membranes resulting in the expression of viral antigens on the myocyte surface [53]. Interestingly, ^18^F-FDG-PET studies have found increased ^18^F-FDG uptake in skeletal muscle infections [54,55]. Infection imaging with ^18^F-FDG-PET is supported based on the fact that granulocytes and mononuclear cells utilize glucose as energy only during their metabolic burst activated by local triggers. Shearer et al. [56], for example, found that inflammatory cells can significantly increase glucose uptake in skeletal muscle.

This raises the question of whether skeletal muscle ^18^F-FDG uptake is altered in post-COVID-19 patients. To date, only one study has investigated this question. Topuz et al. [45] imaged the leg muscles of 68 patients with COVID-19 (mean ± SD age; 56 ± 15 years) and found significantly higher ^18^F-FDG uptake in the psoas muscle during the acute stage of COVID-19 and at a 1-month follow-up. Despite the relatively small sample in this study, their findings indicate that ^18^F-FDG-PET hypermetabolism in skeletal muscles of COVID patients should be carefully monitored and explored in the short- and long-term. As discussed above, fatigue is a significant and key problem in post-COVID-19 patients. Previous studies in patients with other neurological disorders (e.g., multiple sclerosis) have found that increased ^18^F-FDG-PET uptake, interpreted as greater metabolic cost, in skeletal muscles during physical activity might be a mechanism for premature muscle fatigability [57,58,59,60].

## 6. Summary

It is becoming increasingly clear that COVID-19 causes multi-organ impairment with a significant number of patients experiencing post-COVID-19 symptoms. Approximately 70% of post-COVID patients have impairment in one or more organs 4 months after initial COVID-19 symptoms, which has obvious healthcare and public health consequences [61]. Many healthy organs (muscles, lungs, bone marrow, lymphoid tissue, liver, vessels, joints, and organ parenchyma) have elicited greater ^18^F-FDG uptake in post-COVID-19 patients, compared with controls. Importantly, while relative hypermetabolism from multisystem inflammatory syndrome was distinct in some organs, cerebral metabolism studies have only reported regions of hypometabolism, especially in the frontal lobe. This divergence suggests a neuronal/synaptic dysfunction subsequent to inflammatory changes caused by SARS-CoV-2 infection, potentially signifying a substrate for long-term sequelae. Furthermore, the correlation of regions of hypometabolism in patients with persistent increased vascular uptake supports the assumption that brain and whole-body inflammatory changes have different temporal sequences in the disease course [62]. Thus, the potential importance of whole-body ^18^F-FDG-PET/CT in revealing the cerebral and skeletal muscle pathophysiology of long COVID is highly relevant. Overall, ^18^F-FDG-PET has promise for improving post-COVID-19 patient healthcare management, but more in-depth research is needed to describe this potential.

Because ^18^F-FDG-PET/CT has high precision for substantiating localized inflammation at the whole-body level, it would be relatively simple to conduct prospective, longitudinal, multi-center studies in post-COVID-19 patients at different stages of severity. The objective of such studies could be to identify extrapulmonary inflammatory manifestations, search for possible imaging biomarkers, and determine effective treatments of severe and potentially life-threatening inflammatory reactions. Thus, researchers might better identify patients that require specific anti-inflammatory treatments. ^18^F-FDG-PET/CT is a standard for cancer diagnosis and monitoring in most hospitals and clinics throughout the nation and additional usage for research is potentially viable and worthwhile. PET is an accepted, cost-effective tool for a variety of cancer conditions and it is, therefore, likely that this imaging modality will be equally relevant for COVID-19. Lastly, ^18^F-FDG-PET/CT can be a useful adjunct to traditional imaging techniques to help reduce disease burden and provide vital information at the molecular level. In addition, FDG-PET/CT imaging is in alignment with medicine’s shift to a more patient-oriented approach. It has the advantage of allowing histological mapping and a personalized method of treatment. However, further research is necessary to fully explore and validate the effectiveness of FDG-PET/CT imaging as a stand-alone modality in the setting of infectious diseases, such as COVID-19.

## Data Availability

Not applicable.
